# Potential use of multikinase inhibitors in immunosuppressed patients with malignancies including thyroid cancer

**DOI:** 10.1002/cam4.5106

**Published:** 2022-10-06

**Authors:** Neus Basté Rotllan

**Affiliations:** ^1^ Medical Oncology Department, Hospital Clinic Barcelona, Translational Genomics and Targeted Therapies in Solid Tumors August Pi i Sunyer Biomedical Research Institute (IDIBAPS) Barcelona Spain

**Keywords:** immunosuppressed patients, multikinase inhibitors, systemic treatment, thyroid carcinoma

## Abstract

In this article, we focus on a variety of immunosuppression scenarios and whether multikinase inhibitors, as systemic therapy for advanced thyroid carcinoma (TC), could be useful for the treatment of immunocompromised patients with TC. Lenvatinib and sorafenib, among other MKIs, have become the standard of care for advanced TC based on their efficacy data and despite their adverse effects. Currently, published data on MKIs in immunosuppressed patients are scarce. Secondary malignancies can arise in immunosuppressed patients who have undergone solid organ transplantation, human immunodeficiency virus–infected patients, and hematopoietic stem cell transplant recipients. This review will explore different immunosuppression settings, the risk of secondary malignancies in immunosuppressed patients, and the special characteristics of this population. Some considerations regarding anticancer treatment in immunosuppressed patients with advanced malignancies are reviewed.

## Graphical abstract

Multikinase inhibitors such as lenvatinib and sorafenib have become the standard of care for advanced thyroid cancer based on their efficacy. However, published data on multikinase inhibitors in immunosuppressed patients are scarce. Some considerations regarding anticancer treatment in immunosuppressed patients with advanced malignancies are reviewed.

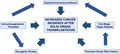

## 1 INTRODUCTION

Thyroid cancer (TC) is the most common endocrine cancer. The incidence of TC has steadily increased over the past decades with no change in mortality rate, which is primarily due to earlier detection.^1^ The incidence in women is approximately triple that in men and the median age at diagnosis is 50 years. Risk factors for the development of TC include radiation and previous exposure to radioactive iodine isotopes.^2^

Surgery is considered the cornerstone treatment for TC, followed by radioiodine in cases of differentiated TC. Systemic treatments are approved for advanced TC (differentiated and medullary) with progressive and symptomatic disease, with high response rates despite significant adverse events (AEs). Multikinase inhibitors (MKIs) are considered the standard therapy for advanced TC.^3^, ^4^, ^5^, ^6^, ^7^ Recently, new drugs with better safety profiles have been developed. Most are considered selective targeted therapies directed toward molecular alterations such as neurotrophic tyrosine kinase and rearranged during transfection receptors.^8^, ^9^, ^10^, ^11^, ^12^, ^13^, ^14^, ^15^, ^16^

Worldwide, immunosuppression includes a wide variety of medical conditions, inter alia solid organ transplantation (SOT), long‐term dialysis due to multiple transfusions, human immunodeficiency virus (HIV) and other infections, autoimmune diseases, bone marrow transplantation, splenectomy, and immunosuppressant therapies, including chronic corticosteroid use.^17^

In this review, we discuss whether different causes of immunosuppression could influence the incidence of TC, survival, and treatment with MKIs in immunocompromised patients with advanced TC and other cancer types. However, it should be noted that there are scarce available data on the immunosuppressed population with advanced TC treated with MKIs.

## 2 HISTORICAL PERSPECTIVE

There are several immunosuppression scenarios that could be associated with a greater risk of cancer. Hematopoietic stem cell transplant (HCT) recipients are at high risk for secondary solid cancers, which increase from 5 years after the procedure, with no plateau over time. In one study, the median time between HCT and the development of secondary TC was 8.5 years.^18^ The risk of cancer increases after HCT for the skin, thyroid, oral cavity, esophagus, liver, nervous system, bone, and connective tissues. Studies investigating the incidence of secondary TC after HCT have shown differing results. However, the Late Effects Working Party of the European Group for Blood and Marrow Transplantation (EBMT) found an increased risk of secondary TC compared with the general population (standardized incidence ratio, 3.26).^18^ Lee et al. reported that secondary TC after HCT had a more aggressive clinical presentation and occurred in younger patients.^19^ Female sex, irradiation history, young age at HCT, and chronic graft‐versus‐host disease (GVHD) were considered risk factors for secondary TC. The 5‐year overall survival rates after diagnosis of solid cancers vary according to the cancer site, with 88%–100% for TC, testis cancer, and melanoma. These rates are similar to de novo cancer rates.^18^, ^20^, ^21^

Patients with HIV are more likely to develop advanced‐stage cancers and to experience increased mortality following a cancer diagnosis, even after adjusting for healthcare‐related factors (suboptimal healthcare, less likelihood of receiving cancer treatment).^22^ Coghill et al. have reported the association between HIV and advanced‐stage cancers at presentation (especially oral cavity, liver, thyroid, breast, prostate, and melanoma) and greater mortality following a cancer diagnosis (tripling mortality for TCs). There appears to be a biological link between HIV‐related immunosuppression and tumor behavior.^23^ Rates of infection‐associated cancers among HIV patients have declined due to the availability of highly active antiretroviral therapy (HAART), although the risk remains higher than for HIV‐uninfected patients.^24^ The types of malignant HIV diseases have changed because of restoration of immunity, resulting in decreasing opportunistic infections, chronicity of HIV infection, a possible oncogenic role of HIV, and aging of the HIV‐infected population.^25^ A French study of HIV‐infected adults treated with HAART found that malignant disease (acquired immune deficiency syndrome [AIDS]‐related or non‐AIDS‐related) was the most common cause of death, accounting for 28%.^25^

The incidence of fatal solid tumors may increase in the future due to increases in life expectancy and the longer duration of HIV seropositivity for HIV‐infected patients treated with HAART.^25^ However, it is uncertain whether HIV independently affects carcinogenesis. Pakkala et al. reported an increased risk of lung cancer in HIV‐infected patients, linked to several factors, including carcinogen exposure (tobacco), immunosuppression, CD4 count, and viral load.^26^ It is important to point out the association between risk factors and cancer development. An in vitro study has shown that the HIV *tat* gene may modulate the expression of certain proto‐oncogenes (c‐myc, c‐fos, and p53) in malignant cells.^27^ The carcinogenic role of HIV in the development of solid malignancies is unknown and may be relatively minor compared with the effect of other risk factors, including tobacco and alcohol use, poor nutrition, coinfection with other viruses (hepatitis, human papillomavirus), and immunodepression, which are often present in patients with HIV, and should be the focus of approaches to prevention and cure.

Patients receiving chronic immunosuppressant therapies such as corticosteroids have experienced endocrine dysfunction as well as those exposed to HCT and total body irradiation (TBI).^28^ However, there are no published data regarding the causality of TC in patients treated with long courses of corticosteroids. Thyroid diseases have been widely associated with HIV infection, although some prevalence reports are controversial. Properzi et al. concluded that the symptomatic thyroid dysfunction rate in well‐treated HIV patients is low when considering age, sex, and T CD4^+^ cell nadir as crucial components of thyroid abnormality.^29^

Candidiasis, a fungal infection, develops in immunocompromised patients due to the ability of these species to adapt to different hosts. A group from Taiwan has reported significantly increased risks for pancreatic, skin, and TC in patients with candidiasis.^30^ This may be, at least partially, related to surveillance bias, especially for skin and TC.

SOT recipients are at high risk for cancer, especially nonmelanoma skin cancers, suggesting that the immune system exercises control over cancer development.^31^ The mechanisms leading to this excess risk in SOT recipients have not been elucidated yet, but some proposals have been explored, such as (i) a direct effect of immunosuppressants on immunosurveillance and the activation of oncogenic viruses, (ii) carcinogenic AEs related to immunosuppressants, (iii) the role of chronic immune system stimulation on cancer development, and (iv) the influence of pre‐existing risk factors in patients with SOT.^32^

In recent decades, the survival of patients after SOT has improved. However, with longer life spans, more SOT patients with functional grafts are at risk of death from other comorbidities, including cancer and cardiovascular disease. SOT recipients are at increased risk of developing cancer three‐ to five‐fold that of the general population, although the reason(s) for the greater risk is unclear.^32^

**FIGURE 1:**
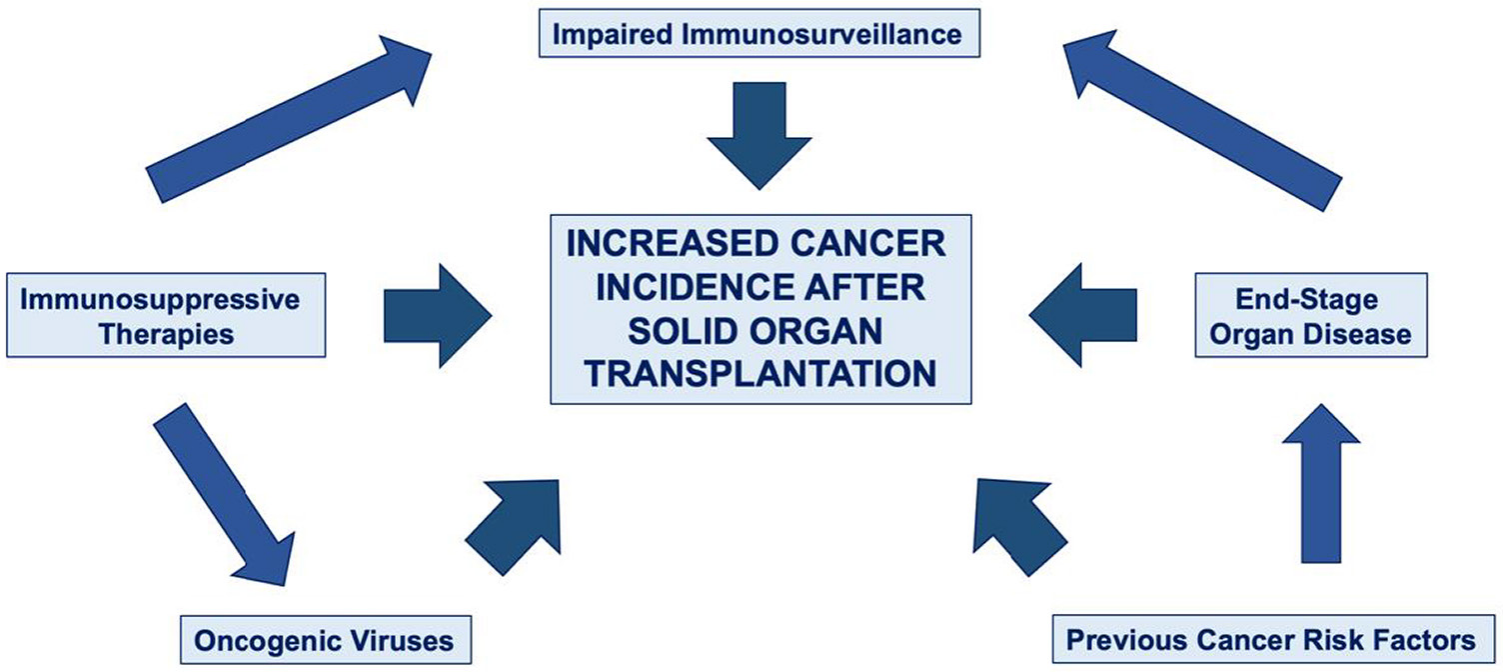
Causes of raised cancer incidence after solid organ transplantation

De novo malignancies in kidney transplant recipients range from 6% to 11% and seem to be more aggressive than among the general population, with low life expectancy.^33^ The mechanism of this excess risk is likely to be multifactorial (Figure 1). While immunosuppression increases the risk of virus‐associated cancers, data on SOT suggest that suppression of immune function also increases the risk of nonviral cancers.

Many studies have investigated the increased incidence of different cancers (nonmelanoma skin cancer, non‐Hodgkin's lymphoma, and malignancies of the lip, kidney, larynx, and thyroid) in the immunosuppressed population (SOT, long‐term dialysis).^31^ Kitahara et al. found a 2.5‐fold greater incidence of TC in transplant recipients (mostly kidney recipients).^34^ Most of the cancers were virus related (Table 1).^35^ As already mentioned, patients with HIV/AIDS have higher incidences of malignancies related to oncogenic viruses.^32^

**TABLE 1:** Association between oncogenic viruses and bacteria and the most frequently seen posttransplant de novo malignancies

Oncogenic viruses and bacteria	Posttransplant de novo malignancies
HPV	Cervix, uterus, vulva, vagina, penis, anus, oropharynx, oral cavity
EBV	Hodgkin's lymphoma, NHL
HHV‐8	Kaposi's sarcoma
HBV, HCV	Hepatocellular carcinoma
Merckel cell polyomavirus	Merkel cell carcinoma
*Helicobacter pylori*	Gastric cancer

Abbreviations: EBV, Epstein–Barr virus; HBV, hepatitis B virus; HCV, hepatitis C virus; HHV‐8, human herpes virus‐8; HPV, human papilloma virus; NHL, non‐Hodgkin's lymphoma.

Thyroid carcinogenesis may be influenced by endocrine and metabolic effects of chronic kidney disease and long‐term dialysis such as impaired DNA repair and decreased benefits of antioxidants due to reduced renal elimination with higher carcinogenic accumulation.^35^, ^36^, ^37^ In the immunocompromised population, TC etiology remains poorly understood, except for the few risk factors already known (female sex, ionizing radiation exposure, obesity). TC does not arise from virus infection‐enriched populations. Immunosuppressor therapies, as prevention of GVHD, do not play a critical role in TC development compared with other virus‐related malignancies.^15^, ^16^, ^17^ Currently, no strong association has been reported between TC and immunosuppressant therapies. Among immunosuppressive maintenance treatments, cyclosporine, and azathioprine were associated with a lower risk of TC than tacrolimus and mycophenolate mofetil.^34^

There are no published studies of immunocompromised patients with de novo advanced TC given MKIs. Possibly, close follow‐up helps to detect TC at an early stage when surgery is indicated for these patients.

## 3 CURRENT SITUATION

Angiogenesis is considered a hallmark for tumor development and progression. Neovasculature is tortuous and disorganized, enhancing vascular permeability, which leads to increased interstitial fluid pressure and reduced blood perfusion and oxygenation. Tissue hypoxia can trigger the expression of multiple growth factors by cancer and stromal cells (i.e., fibroblasts, macrophages) recruited to tumors inducing an immunosuppressive tumor microenvironment.^38^ Antiangiogenic therapies can initially normalize vascular permeability and reduce tumor angiogenesis. Intrinsic or adaptive resistance induces tumor hypoxia and immunosuppression. Different mechanisms of resistance to antiangiogenics include (i) alternative angiogenic pathways activation (vascular endothelial growth factor‐independent manner), (ii) recruitment of bone marrow‐derived cells (M2 tumor‐associated macrophages, myeloid‐derived suppressor cells, regulatory T cells) and cancer‐associated fibroblasts promoting tumor growth and epithelial‐to‐mesenchymal transition, and (iii) participation of malignant cells in neovascularization (vasculogenic mimicry, vasculogenesis, or splitting angiogenesis).^38^ MKIs are considered the standard therapy for advanced TC.^3^, ^4^, ^5^, ^6^, ^7^

There are limited data relating to the management of advanced TC with MKIs in the immunosuppressive context, such as SOT recipients, HCT recipients, HIV/AIDS patients, and those receiving immunosuppressant therapies, among others. However, there is some relevant information that should be considered.

Pharmacokinetic risk is relevant in HIV‐infected cancer patients receiving concomitant HAART and anticancer systemic treatments due to the potential for drug–drug interactions, leading to inhibition of drug transporters and metabolizing enzymes (ABCB1 and cytochrome P450), potentially resulting in severe toxicity. Most MKIs are primarily metabolized by cytochrome P450 (isoform CYP3A4) and have a narrow therapeutic index. Protease inhibitor‐based HAART regimens (e.g., ritonavir) can result in drug–drug interactions, as evidenced by taxanes.^39^ Thus, they should not be given to patients receiving MKIs. Uldrick et al. evaluated drug–drug interactions in patients with Kaposi sarcoma treated with sorafenib (a vascular epithelial growth factor receptor, c‐kit, and platelet‐derived growth factor receptor‐targeted treatment) and ritonavir (HAART with strong CYP3A4 inhibitory activity). Strong CYP3A4 inhibition may influence sorafenib toxicity. Therefore, alternative HAART without known sorafenib interactions should be administered when the two drug regimens are given concurrently.^40^ However, Loulergue et al. evaluated concurrent administration of raltegravir‐based HAART and MKIs in patients with HIV with advanced, non‐AIDS‐defining malignancies, and found a promising safety profile.^41^ Based on this observation, HAART raltegravir‐containing regimens could be safe for administration to patients treated with MKIs.

Another issue considered is the immunosuppressive characteristics of some MKIs. Immunosuppression is due to an imbalance between regulatory T cells and CD8+ T cells. Preclinical data with sorafenib have shown a dose‐ and time‐dependent immunomodulatory effect. A larger sorafenib dose has been shown to have immunosuppressive activity.^42^ However, there is no clinical evidence of immunosuppression reported in patients with advanced TC treated with MKIs.

Even if no definitive conclusions can be drawn for immunocompromised patients with advanced TC, there are some data that provide a wide view of immunosuppression setting. Tumor recurrence is the most important limiting factor for the long‐term survival of patients with SOT. Recurrence of hepatocellular carcinoma (HCC) postliver SOT has been described in around 16–21% of patients according to different multidisciplinary treatment approaches.^43^, ^44^, ^45^, ^46^, ^47^ Yang et al. have shown that survival improves significantly in certain patients after aggressive surgery for recurrence (20.9 months compared with 9.4 months for unresectable recurrent HCC treated with nonsurgical therapy or 2.4 months for best supportive care [BSC]). Conservative therapies included MKIs (67%), systemic chemotherapy (42%), transarterial chemoembolization (22%), radiotherapy (17%), and BSC (15%).^47^ Unfortunately, no data on dosing and toxicities for MKIs (regorafenib, sorafenib, and lenvatinib) used in this setting were shown. However, other reports have presented sorafenib AEs, including hand‐foot skin reaction (60%), diarrhea (40%), and fatigue (17%), with dose reduction (400 mg/day) in over 50% of patients, and withdrawal in cases of renal and hepatic insufficiency. Therefore, sorafenib usually starts at a dose of 400 mg/day in this patient group.

As previously mentioned, the higher incidence of AEs in HIV patients may be associated with pharmacokinetic and/or pharmacodynamic interactions with immunosuppressants, and metabolic pathway overlaps. The post‐SOT AE profile of sorafenib combined with immunosuppressors is of concern, but there are no conclusive data.^48^ Sirolimus, an mTOR inhibitor used as immunosuppressant therapy, resulted in improved survival after HCC recurrence compared with calcineurin inhibitor therapy.^47^ mTOR inhibitors are thought to protect from de novo malignancies and HCC recurrences compared with calcineurin inhibitors, which have a greater risk of post‐SOT recurrence.^44^ The intention is to maintain the immunosuppression therapy as low as possible to reduce the anticancer immune activity. Published data have shown that sorafenib in combination with sirolimus does not result in a drug–drug interaction, and does not need dose adjustment compared with other regimens.^48^ Meanwhile, a systematic review from De'Angelis et al. reported high toxicity rates, with four deaths, with the combination of an immunosuppressor such as everolimus and sorafenib. Dose reduction (42.1%) and treatment discontinuation (10%) were observed in most of the studies. Sorafenib mainly resulted in stable disease (46.5%), although a large number of patients (37%) presented with disease progression.^43^ Therefore, considering that the cost‐effectiveness and risk–benefit of sorafenib are currently unknown, further investigation into this population is needed.

## 4 TREATMENT RECOMMENDATIONS

As previously mentioned, MKIs are the standard therapy for advanced TC.^3^, ^4^, ^5^, ^6^, ^7^ Based on published data, any systemic treatment can be proposed for immunosuppressed patients with advanced TC. However, some recommendations can be made in the context of immunosuppression and the use of MKIs.

All patients receiving HCT should be informed of the risk of secondary cancers and encouraged to undergo regular screening based on their risk profile. The Late Effects Working Party of the EBMT^18^ recommends physical examination (neck palpation) annually, with no clear evidence for routine imaging at screening. However, in addition to regular follow‐up, Lee et al. recommend ultrasound examination of the neck, as that may result in earlier detection and treatment.^19^ Patients who are most at risk include long‐term pediatric survivors, women, those who have undergone myeloablative TBI, and those with chronic GVHD.

Among HIV‐infected patients, systematic screening for tobacco and alcohol misuse is advised, along with screening for lung carcinoma (yearly X‐ray) and cervical and rectal cancer (systematic examination, cervical smears, and human papillomavirus detection).^25^ In the future, the occurrence of fatal solid tumors may increase because of increased life expectancy and longer duration of HIV seropositivity for patients with HIV infection treated with HAART. Concomitant HAART and anticancer systemic treatments present a higher risk of pharmacokinetic interaction, although raltegravir‐based HAART could be a safe option for HIV‐infected patients treated with MKIs.^41^

In case of malignancy recurrence post‐SOT, such as HCC, different MKIs (sorafenib, lenvatinib, and regorafenib) have been explored as monotherapies and in combination with mTOR inhibitors, and have demonstrated improved outcomes compared with BSC.^43^ A synergistic effect has been suggested for concomitant mTOR inhibitor and sorafenib, but there are inconclusive data thus far.^49^ The MKI toxicity profile was significant, requiring dose reduction in many patients. Pharmacokinetic and/or pharmacodynamic drug–drug interaction may be responsible for the high rate of AEs.^27^ However, mTOR inhibitors seem to have good immunosuppressive activity with concomitant antitumor properties and may protect from de novo malignancies and/or prevent HCC recurrence.^32^ mTOR inhibitors have been suggested as immunosuppressant therapy post‐SOT with the aim of controlling or delaying graft rejection and tumor recurrence risk.

## 5 DISCUSSION

The immunosuppressant scenario includes a wide spectrum of conditions such as HCT, HIV/AIDS, other infections, SOT, long‐term dialysis, and immunosuppressant therapies, including chronic corticosteroid use. In these patients, regular follow‐up may help with the early detection of malignancies and treatment with curative intent. Due to the increased risk of secondary malignancies in patients receiving HCT, screening is highly recommended, including systematic screening for lung, rectal, and cervical cancer in patients with HIV infection.^18^

MKIs are considered standard therapy for many malignant tumor types including TC.^3^, ^4^, ^5^, ^6^, ^7^ Despite their beneficial effect, MKIs are associated with AEs, with a narrow therapeutic index. The combination of an MKI with cytochrome P450 inducers will lead to severe toxicities due to drug–drug interaction.^39^, ^40^, ^41^ Immunocompromised patients frequently experience this situation, especially when combining anticancer systemic treatments such as MKIs and HAART in HIV‐infected patients, or immunosuppressor agents in patients with recurrences post‐SOT. In the case of the HIV‐infected population, HAART regimens including raltegravir could be safely administered to patients treated with MKIs. Meanwhile, MKIs and tapered doses of immunosuppressants, as protection against SOT rejection, could be an option for unresectable recurrence of HCC. Furthermore, mTOR inhibitors are associated with antitumor potential, which may be beneficial for tumor control.

To date, there are no data on managing immunosuppressed patients with advanced TC who are amenable to systemic treatment. Current published reports of TC patients mainly focus on the treatment of local disease with curative intent and the characteristics of immunosuppression.^50^, ^51^, ^52^ Any conclusions are based on different immunosuppression scenarios. So far, the management of immunocompromised patients with advanced malignancies remains a challenge for clinicians.

## AUTHOR CONTRIBUTION

The author has written and completed the manuscript, including the table and figure.

## FUNDING INFORMATION

The author received an honorarium payment from Eisai Farmacéutica SA in line with ICMJE guidelines.

## CONFLICT OF INTEREST

The author has received research honoraria, and nonfinancial or other support from BioNTech, Bristol‐Myers Squibb, Debiopharm, Eisai, ISA Therapeutics, Merck Serono, MSD, Bayer, and Roche.

## ETHICAL STATEMENT

Not Applicable.

## Data Availability

From published data.
